# A novel motion-reconstruction method for inertial sensors with constraints

**DOI:** 10.1007/s11044-022-09863-8

**Published:** 2022-12-08

**Authors:** Rene Neurauter, Johannes Gerstmayr

**Affiliations:** Department of Mechatronics, University of Innsbruck, Technikerstraße 13, Innsbruck, 6020 Austria

**Keywords:** Multibody system dynamics, Measurement unit, Optimization, Strapdown inertial navigation, Orientation estimation, Sensor calibration

## Abstract

Motion reconstruction for rigid bodies and rigid-body frames using data from inertial measurement units (IMUs) is a challenging task. Position and orientation determination by means of IMUs is erroneous, as deterministic and stochastic errors accumulate over time. The former of which errors can be minimized by standard calibration approaches, however, sensor calibration with respect to a common reference coordinate system to correct misalignment, has not been fully addressed yet. The latter stochastic errors are mostly reduced using sensor fusion. In this paper, we present a novel motion-reconstruction method utilizing optimization to correct measured IMU data by means of correction polynomials to minimize the deviation of motion constraints. In addition, we perform gyrometer and accelerometer calibration with an industrial manipulator to address deterministic IMU errors, especially misalignment. To evaluate the performance of the novel methods, two types of experiments, one at constant orientation and another with simultaneous translation and rotation, were conducted utilizing the manipulator. The experiments were repeated for five individual IMUs successively. Application of the calibration and optimization methods yielded an average decrease of 95% in the maximum position error compared to the results of common motion reconstruction. Moreover, the average position error over the measurement duration decreased by nearly 90%. The proposed method is applicable to velocity, position, and orientation constraints for every experiment that starts and ends at standstill.

## Introduction

Reconstruction of rigid-body motion by means of measurement data acquired with a low-cost strapdown inertial measurement unit (IMU) is limited to measurements with a short time duration, as position errors due to sensor drifts and erroneous sensor axes increase quadratically with time [1]. Thus, a trend toward sensor fusion has emerged to address sensor drifts in recent years. In addition, various calibration approaches to correct the sensor axes were proposed. However, little research has investigated the potential of constraints in the context of motion reconstruction. This paper contributes a novel method for motion reconstruction utilizing motion constraints to optimize measured quantities, such that the deviation in computed position and orientation is minimized.

Conventional strapdown IMUs are cheap and therefore applied in many fields for motion tracking, e.g., medicine [2], robotics [3], and sports [4]. In addition, IMUs are utilized in life-threatening natural hazards to measure the internal dynamics, e.g., rockfalls [5] and snow avalanches [6, 7]. Conventional IMUs are composed of three sensors; an accelerometer, a gyrometer, and a magnetometer, measuring spatial translational acceleration, angular velocity, and magnetic-flux density, respectively. Each sensor measures with respect to an individual coordinate system defined by three unit vectors ($x, y, z$) that are mutually perpendicular to one another in the right-hand sense. Ideally, the three coordinate systems coincide, such that there is a common origin, and the individual $x$-, $y$-, and $z$-axes are parallel, point in the same direction, and are of equal length. However, this assumption is not valid due to deterministic errors, which are nonorthogonality, misalignment, and wrong scaling of the sensor axes.

Nevertheless, there are calibration approaches to minimize deterministic errors. For accelerometer calibration, there is the well-established, so-called six-position calibration, e.g., used in [8, 9], which denotes calibration in six defined orientations. Syed et al. proposed an attempt, where 18 positions are used for calibration, which had no significant advantage compared to the six-position calibration [10]. Gyrometer calibration is performed through defined rotations and differs in the calibration domain, which is either angle or angular velocity. In the former domain, computed angles from IMU measurements are compared with a reference angle from an experiment with a known angle of rotation. In the latter domain, the measured angular velocity is compared with a reference angular velocity from an experiment with constant angular velocity. However, few researchers have proposed calibration methods in the angular-velocity domain, as it requires special equipment to provide constant angular velocity. The misalignment of the individual sensors is a still-existing problem in the field of IMU calibration. Zhang et al. mention that the misalignment of the individual sensors can be corrected if the calibration of all sensors is performed by means of a single system (e.g., a robot [11]), which, however, was not addressed in their work [12].

Regardless of IMU quality or calibration, methods for inertial-motion reconstruction were developed. Specifically, methods to compute orientations via time integration of angular velocities and to compute positions by double time integration of accelerations, which are first transformed into a global frame by means of computed orientations. The first approaches of orientation estimation from strapdown inertial measurements were proposed by Bortz [13]. Savage covers fundamental inertial-navigation concepts regarding orientation and position estimation in [14, 15]. More recently, the computation of orientation is performed utilizing Lie-group methods [1, 16]. Lie-group methods, combined with standard time-integration schemes for the translational part, represent the state-of-the-art in motion reconstruction, as they are free of singularities and result in high accuracy.

The deviations in computed position and orientation arise from deterministic and stochastic errors in IMU measurement data. The former errors are addressed with the mentioned calibrations. The latter errors, foremost sensor drift, remain, as the elimination is not possible due to its randomness [17]. Hence, numerous scholars have conducted research on sensor fusion employing Kalman filters [18, 19]. On the one hand, there is the field of IMU sensor fusion, where the estimated orientation derived from accelerometer and magnetometer data is utilized to correct orientation computed by means of measured angular velocities [20, 21]. On the other hand, multisensor fusion is studied, where, e.g., the computed position is corrected utilizing a global navigation satellite system (GNSS) [22, 23]. As Kalman filtering requires tuning of various parameters, Madgwick proposed an approach toward simplification [24], further resulting in increased computational efficiency without accuracy loss [25].

A completely different, rarely investigated approach is based on optimization with constraints. There is a well-established approach in pedestrian tracking, known as zero-velocity update [26, 27], where the fact that one foot is stationary at a time while walking is used as a constraint. Recently, the authors of the present paper proposed a similar approach, where acceleration is optimized, such that after time integration, the velocity at the end of motion equals zero [16].

This paper contributes toward motion reconstruction by means of optimization with respect to constraints and toward precise calibration. A novel method corrects measured accelerations and angular velocities by means of polynomial functions to minimize the deviation in constraints of the derived velocity, position, and orientation. In addition, a precise calibration for the accelerometer and gyrometer is performed, such that misalignment between sensors is corrected by utilizing an industrial robot for manipulation during calibration measurements. The motion reconstruction and optimization are applied to measurement data from five individual IMUs of the same type. These five IMUs were successively clamped onto the robot, calibrated, and then utilized to measure two different motions, i.e., a trajectory with motion at constant orientation and another trajectory covering simultaneous translation and rotation, with a duration of 23 seconds each. Results prior to the optimization as well as optimized data are compared to a reference trajectory provided by the robot controller to evaluate the performance of the methods.

The algorithms and methods developed in this paper can be applied to any situation where velocity, position, and orientation at the start and end are either fully or partly known. Nevertheless, the long-term objective of this work is the motion reconstruction of particles in snow avalanches. However, in snow avalanches, there is no reference data to evaluate the methods and algorithms presented in the following. Therefore, experiments utilizing an industrial manipulator may be a good start to meet this long-term objective.

## Motion reconstruction

In many fields, e.g., vehicle navigation or satellite-attitude estimation, motion reconstruction is utilized for real-time prediction of trajectories. In this work, however, trajectories are computed as part of the postprocessing using IMU measurement data, which are translational acceleration, angular velocity, and magnetic-flux density. However, the magnetic field is not considered in the present work. Note that all methods are applied to calibrated accelerations and angular velocities. The latter calibrations are described in Sect. 4.

Motion reconstruction by means of IMU data can be split into a rotational part, computing rotation matrices, and a translational part, computing velocity and position, whereas the entire equations describing motion are denoted as the equations of motion (EOM).

### Frame transformations

Within motion-reconstruction, data is represented in different frames, indicated by left superscripts. There is the sensor frame (S), which is attached to the IMU and thus to the measuring system. Since the measuring system is moving and the acquired data is discretized, the pose of the measuring system is given by sensor frames ($\mathrm{S}_{i}$) for $n$ measured time steps $i$, corresponding to time $t_{i}$, with $i \in \{0,1,2, \ldots , n\}$. Furthermore, there is the initial frame (I), which corresponds to the initial sensor frame ($\mathrm{S}_{0}$). For coordinate transformation (passive rotation) from the $i$th sensor frame ($\mathrm{S}_{i}$) to the initial frame (I), we introduce the transformation

1 $$ ^{\textrm {I}}\mathbf{u}_{i} = \mathbf{R}_{i} \ {^{{\textrm {S}_{i}}}\mathbf{u}_{i}} , $$

where $\mathbf{u}$ is a placeholder for translational acceleration, velocity, or position and $\mathbf{R}$ is a rotation matrix to perform the transformation. As ${}^{\textrm {I}}\mathbf{u} = {{{}^{\mathrm{S}_{0}}} \mathbf{u}}$, there is the special case of $\mathbf{R}_{0} = \mathbf{I}_{3}$, where $\mathbf{I}_{3} \in \mathbb{R}^{3\times 3}$ denotes the identity matrix.

### Computation of rotation

To obtain the orientation of the measuring system and to allow a transformation of translational data from the sensor frame to the initial frame, rotation matrices have to be computed by means of measured angular velocities ${}^{\textrm {S}}\pmb{\omega} \in \mathbb{R}^{3\times n}$ and a start orientation $\mathbf{R}_{0}$.

Thus, to determine the rotation matrix $\mathbf{R}$ ∈ SO(3), it is required to solve the kinematic reconstruction equation [28, 29]

2 $$ \dot{\mathbf{R}}=\mathbf{R} \ {^{\textrm {S}}{ \mathbf{\tilde{\pmb{\omega}}}}}, $$

where $\dot{\mathbf{R}}$ is the time derivative of ${\mathbf{R}}$ and $^{\textrm {S}}\tilde{\pmb{\omega}}$ ∈ $\mathfrak {so}(3)$ describes the skew symmetric matrix of angular velocities $\pmb{\omega}$ in the sensor fixed frame, such that $\pmb{\omega} \times \mathbf{y} = \mathbf{\tilde{\pmb{\omega}}} \mathbf{y}$ for $\pmb{\omega}, \mathbf{y} \in \mathbb{R}^{3}$ [30]. To derive a solution of Eq. (2), the well-established approach [31, 32]

3 $$ \mathbf{R}_{i+1}= \mathbf{R}_{i} \exp (^{\textrm {S}_{i}} \tilde{\pmb{\Omega}}) $$

is applied, with the terms incremental rotation vector $^{\textrm {S}_{i}}{\pmb{\Omega}}$, see Eq. (6), and Euler–Rodrigues formula [33]

4 $$ \textrm {exp}(\tilde{\pmb{\Omega}}) = \mathbf{I}_{3} + \mathrm{sinc} ( \Vert{\pmb{\Omega}}\Vert ) \tilde{\pmb{\Omega}}+ \frac{1}{2} \mathrm{sinc} ^{2} \left (\frac{\Vert{\pmb{\Omega}}\Vert}{2}\right ) \tilde{\pmb{\Omega}}^{2}, $$

with the cardinal sine function [34]

5 $$ \mathrm{sinc}(\Vert{\pmb{\Omega}}\Vert ) = \textstyle\begin{cases} 1 & \text{if } \Vert{\pmb{\Omega}}\Vert = 0 \\ \dfrac{\sin \Vert{\pmb{\Omega}}\Vert}{ \Vert{\pmb{\Omega}}\Vert } & \text{else}. \end{cases} $$

**Fig. 1:**
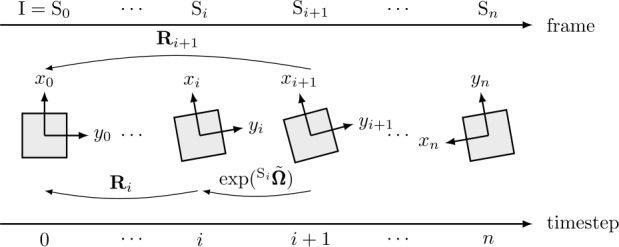
Frames according to time steps and transformation between different coordinate systems is shown on the inplane rotation case. Utilizing the exponential map on incremental rotations $\tilde{\pmb\Omega}$ derives the rotation between two subsequent frames

The Euler–Rodrigues formula, see Eq. (4), is a common approach to compute the exponential map, thus mapping elements of $\mathfrak {so}(3)$ into SO(3). Hence, the exponential map in Eq. (3) can be interpreted as an active rotation between two successive orientations, see Fig. 1. At this point, other maps could be used instead of the exponential map, e.g., the Cayley map, as proposed in [6]. The incremental rotation vector $^{\textrm {S}_{i}}\pmb{\Omega}$ is given by [35]

6 $$ ^{\textrm {S}_{i}}{\pmb{\Omega}}=\frac{1}{2} \ \Delta \,t \ ({^{\textrm {S}_{i}}{ \pmb{\omega}}_{i}}+{^{\textrm {S}_{i}}{\pmb{\omega}}_{i+1}}), $$

applying the trapezoidal integration rule to approximate Eq. (3) in Lie algebra. The time step size $\Delta t$ for two consecutive time steps $t_{i}$ and $t_{i+1}$ is defined by

7 $$ \Delta t = t_{i+1} - t_{i}. $$

Note that in Eq. (6), the angular velocities ${\pmb{\omega}}_{i}$ and ${\pmb{\omega}}_{i+1}$ are represented in the common frame ${\textrm {S}_{i}}$. However, ${{\pmb{\omega}}_{i+1}}$ is measured in frame $\mathrm{S}_{i+1}$ and therefore needs to be transformed into frame $\mathrm{S}_{i}$ via

8 $$ {^{\textrm {S}_{i}}{\pmb{\omega}}_{i+1}}=\exp (^{\textrm {S}_{i}} \tilde{\pmb{\Omega}})\,{^{\textrm {S}_{i+1}}{\pmb{\omega}}_{i+1}}. $$

Thus, the rotation vector ${^{\textrm {S}_{i}}{\pmb{\Omega}}}$ from Eq. (6) is computed iteratively, as substituting Eq. (8) into Eq. (6) derives the implicit equation

9 $$ {^{\textrm {S}_{i}}{\pmb{\Omega}}_{k+1}}=\frac{1}{2} \ \Delta \,t \ ({^{ \textrm {S}_{i}}{\pmb{\omega}}_{i}}+\exp (^{\textrm {S}_{i}} \tilde{\pmb{\Omega}}_{k})\,{^{\textrm {S}_{i+1}}{\pmb{\omega}}_{i+1}}) $$

for $k \in \{0,1,2,\ldots,m\}$ iterations. As the start value for ${^{\textrm {S}_{i}}\pmb{\Omega}_{k=0}}$ in Eq. (9),

10 $$ ^{\textrm {S}_{i}}{\pmb{\Omega}}_{0}=\frac{1}{2} \ \Delta \,t \ ({^{ \textrm {S}_{i}}{\pmb{\omega}}_{i}}+{^{\textrm {S}_{i+1}}{\pmb{\omega}}_{i+1}}) $$

is applied. The number of iterations $m$ is determined through the convergence criterion

11 $$ \Vert {^{\textrm {S}_{i}}\pmb{\Omega}_{k+1}} - {^{\textrm {S}_{i}} \pmb{\Omega}_{k}} \Vert _{1} < 1 \cdot 10^{-15}. $$

For the experiments in this paper, iterations were in the range of 3–7 depending on the magnitude of the measured angular velocities. This is consistent with the fact that higher angular velocity leads to a larger difference in two subsequent orientations at constant sampling frequency. Note that Eq. (10) generally provides a good approximation for Eq. (6) and can therefore be used to speed up the computation at the cost of minor errors in the derived rotations. Additionally, the algorithm could be initialized with ${}^{\textrm {S}_{i}}{\pmb{\Omega}}_{0}=[0\ 0\ 0]^{\mathrm{T}}$ to simplify the algorithm at the cost of one additional iteration.

### Computation of velocity and position

In this section, translational velocities and positions are derived by means of time integration of measured accelerations. Due to the technology of strapdown IMUs, measured accelerations are the sum of accelerations from nongravitational forces and gravitational forces [14]. In contrast to measured accelerations of the moving measuring system, however, the gravity vector is time invariant and therefore constant with respect to the initial frame. Apparently, the gravity vector has to be eliminated to obtain accelerations, which describe the translational motion of the measuring system.

The most common way to eliminate gravity is to transform measured accelerations ${}^{\textrm {S}}\mathbf{a} \in \mathbb{R}^{3\times n}$ into the initial frame, where gravity is constant, and subtract gravity from accelerations ${}^{\textrm {I}}\mathbf{a}$. This transformation can be performed utilizing the rotation matrices derived in Sect. 2.2, yielding

12 $$ ^{\textrm {I}}\mathbf{a}^{*} = {^{\textrm {I}}\mathbf{a}} - {^{\textrm {I}} \mathbf{g}} = \mathbf{R} \ {^{\textrm {S}}\mathbf{a}} - {^{\textrm {I}} \mathbf{g}}, $$

where ${^{\textrm {I}}\mathbf{g}}$ is the gravity^1^ vector pointing toward the center of the earth. Time integration of gravity-eliminated accelerations from Eq. (12) derives

13 $$ ^{\textrm {I}}\mathbf{v}(t)=\int _{t} \left ( \mathbf{R}(\tau ) {^{ \textrm {S}}\mathbf{a}(\tau )} - {^{\textrm {I}}\mathbf{g}}\right ) \mathrm{d}\tau , $$

14 $$ ^{\textrm {I}}\mathbf{p}(t)= \int _{t} \left ( \int _{\tau _{2}}\left ( \mathbf{R}(\tau _{1}) {^{\textrm {S}}\mathbf{a}(\tau _{1})}- {^{\textrm {I}} \mathbf{g}}\right ) \mathrm{d}\tau _{1}\right ) \mathrm{d}\tau _{2}, $$

defining translational velocity $^{\textrm {I}}\mathbf{v}$ and position $^{\textrm {I}}\mathbf{p}$ with respect to the initial frame (I), respectively. Computation of the latter time integrals utilizing the well-known explicit Euler method yields

15 $$\begin{aligned} ^{\textrm {I}}\mathbf{v}_{i+1}&={^{\textrm {I}}\mathbf{v}_{i}}+\Delta \,t\, \, (\mathbf{R}_{i} {^{\textrm {S}}\mathbf{a}}_{i} - {^{\textrm {I}} \mathbf{g}}), \end{aligned}$$

16 $$\begin{aligned} ^{\textrm {I}}\mathbf{p}_{i+1}&={^{\textrm {I}}\mathbf{p}_{i}}+\Delta \,t\, \, {^{\textrm {I}}\mathbf{v}}_{i}, \end{aligned}$$

for translational velocity and position, respectively.^2^

**Fig. 2:**
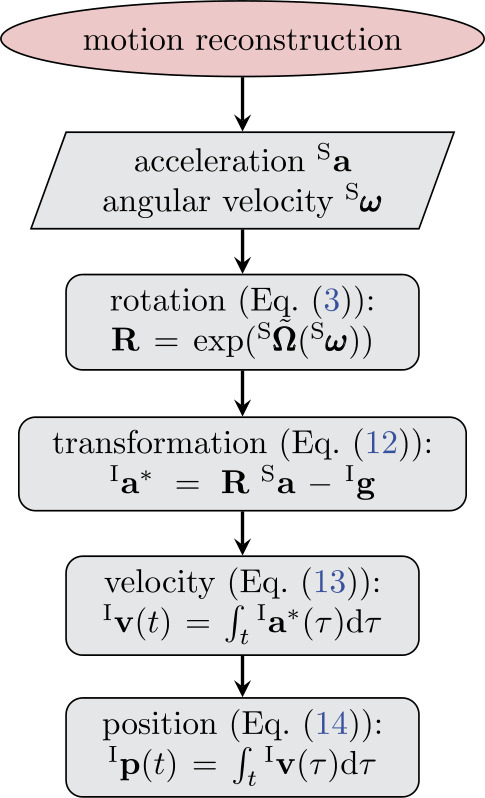
Overview of the motion reconstruction that derives position $\mathbf{p}$ from calibrated acceleration ${\mathbf{a}}$ and angular velocity ${\pmb{\omega}}$, see Sect. 4 (Color figure online)

In summary, Sect. 2 derives the theoretical framework to compute velocity, position, and orientation from measured acceleration and angular velocity. An overview of the motion-reconstruction method, derived in this section, is shown in Fig. 2.

## Optimization of position and orientation

Although an IMU is calibrated, errors in computed positions are still increasing quadratically, foremost due to accelerometer sensor drifts. Thus, without addressing these errors, only short-time IMU measurements are significant [37]. Fortunately, optimization is possible due to constraints, which, however, differ for various applications of IMUs, e.g., pedestrian trackers [27] and snow avalanches [16]. In this paper, we investigate motion that starts and ends at standstill, resulting in physical constraints regarding translations and rotations.

The purpose of the optimization (specifically a minimization) presented in the following, is to correct measured acceleration and angular velocity by means of polynomial functions to derive velocity, position, and orientation, such that the error in constraints is minimized. Hence, we do not utilize constrained optimization [38], but an optimization algorithm to minimize errors in constraints seeking the optimal coefficients for the defined correction polynomials. The degrees of freedom (DOF), and thus the number of polynomial terms, are defined by the number of constraints, as one spatial constraint adds 3 DOF to the EOM. In the case of simultaneous translation and rotation, the latter lead to nonlinear EOM, see Eq. (14), which includes Eq. (3) and Eq. (4). To solve the nonlinear EOM we utilize the well-known Nelder–Mead algorithm [39], which is available in Scipy [40], a common scientific library for Python. Additionally, we investigate a simplified case and solve it analytically. The simplified case results from pure translational motion at constant orientation, thus without performing any rotations, yielding linear EOM. The analytical solution is further used to validate the formulated minimization problem, see Sect. 6. It should be noted that application of the following optimization method requires an initial solution for velocity, position, and orientation derived from the motion-reconstruction method, see Sect. 2.

### Constraints and correction polynomials

The measurements performed in this work always start and end at standstill, thus enabling constraints for either measured or computed rotational and translational quantities. Neglecting the Earth’s motion, a standstill IMU should solely measure gravitation by means of the accelerometer and no angular velocity at all by means of the gyrometer. These constraints are partially considered with a calibration, see Sect. 4. However, constraints on acceleration and angular velocity level cannot be satisfied, as these quantities are subject to optimization, to meet constraints on translational velocity, position, and orientation level, which will be described in the following.

#### Rotational constraints

We already defined ${\mathbf {R}_{\mathrm{0}}}= \mathbf{I}_{3}$ in Sect. 2.1, as at time step $i$=0 no rotations were performed yet. At time step $i$=$n$, however, the computed orientation ${\mathbf {R}_{n}}$ differs from a reference orientation ${\mathbf {R}_{\mathrm {ref},n}}$ due to integration errors and sensor errors. Hence,

17 $$ \Delta \mathbf{R} = {\mathbf {R}_{\mathrm {ref},n}}\ {{\mathbf {R}_{n}}^{\mathrm{T}}} $$

denotes the error in orientation. For minimization purposes, this error in orientation is further expressed as an error in angles via the matrix logarithm [41], yielding

18 $$ \Delta \tilde{\pmb{\theta}} = \mathrm{log}(\Delta \mathbf{R}). $$

To satisfy the constraint^3^

19 $$ {\mathbf {R}_{n}}\overset{!}{=} {\mathbf {R}_{\mathrm {ref},n}}, $$

we seek the simplest spatial polynomial with three parameters, hence a constant correction term ${_{\omega}\mathbf {c}}$, which corrects angular velocity. In this work, the reference orientation ${\mathbf {R}_{\mathrm {ref},n}}$ is provided by means of the robot controller. However, in field experiments, orientation can be determined with the help of an accelerometer and a magnetometer performing a so-called Earth-frame transformation [42, 43].

#### Rotational correction polynomial

Correction of angular velocity, such that the computed rotation at time step $n$ satisfies Eq. (19), is performed by means of a constant term ${_{\omega}\mathbf {c}}$, yielding

20 $$ {^{\textrm {S}_{i}}{\pmb{\omega}}_{\mathrm{opt},i}} = {^{\textrm {S}_{i}}{ \pmb{\omega}}_{i}} + {_{\omega}\mathbf {c}}, \ \forall i \in \{0,1,2,\ldots,n\}, $$

where ${^{\textrm {S}_{i}}{\pmb{\omega}}_{\mathrm{opt}}}$ and ${^{\textrm {S}_{i}}{\pmb{\omega}}}$ are optimized and calibrated angular velocity, respectively.

#### Translational constraints

For a standstill IMU after a performed measurement we denote the constraint

21 $$ {^{\textrm {I}}{\mathbf {v}_{n}}} \overset{!}{=} 0, $$

as a nonmoving rigid body has zero velocity, yielding an error of $\Delta\mathbf{v}$ at the end of motion,

22 $$ \Delta\mathbf{v}={^{\textrm {I}}{\mathbf {v}_{n}}}. $$

Due to the known position by means of the robot controller (or GNSS in outdoor experiments), a further constraint

23 $$ {^{\textrm {I}}{\mathbf {p}_{n}}} \overset{!}{=} {^{\textrm {I}}{\mathbf {p}_{\mathrm {ref},n}}} $$

is introduced, since the calculated position ${\mathbf {p}_{n}}$ must equal the position according to the reference ${\mathbf {p}_{\mathrm {ref},n}}$ provided by the robot controller (or GNSS in outdoor experiments). This leads to a position error at the end of motion according to

24 $$ \Delta\mathbf{p}={^{\textrm {I}}{\mathbf {p}_{n}}} - {^{\textrm {I}}{\mathbf {p}_{\mathrm {ref},n}}}. $$

Of course, the constraints from Eq. (21) and Eq. (23) are also valid for IMU data prior to the measurement. However, as sensor errors are zero due to a bias correction at the beginning of the measurement, the constraints

25 $$\begin{aligned} {^{\textrm {I}}{\mathbf {v}_{\mathrm{0}}}} &\overset{!}{=} 0, \end{aligned}$$

26 $$\begin{aligned} {^{\textrm {I}}{\mathbf {p}_{\mathrm{0}}}} &\overset{!}{=} {^{\textrm {I}}{\mathbf {p}_{\mathrm{ref,0}}}}, \end{aligned}$$

are already met by proper calibration.

#### Translational correction polynomial

To satisfy the constraints on translational velocity and position, respectively, Eq. (21) and Eq. (23), we add a polynomial

27 $$ {_{\mathrm{a}}\mathbf {c}}_{i} = {_{0}\mathbf {c}}+ {_{1}\mathbf {c}}\ t_{i}, $$

with six parameters to measured accelerations ${^{\textrm {S}}{\mathbf{a}}_{i}}$, yielding optimized accelerations

28 $$ {^{\textrm {S}}{\mathbf{a}}_{\mathrm{opt},i}} = {^{\textrm {S}}{\mathbf{a}}_{i}} + {_{\mathrm{a}}\mathbf {c}}_{i}, \ \forall i \in \{0,1,2,\ldots,n\}, $$

where ${_{\mathrm{a}}\mathbf {c}}$ is the correction polynomial with a coefficient ${_{0}\mathbf {c}}$ for the constant term and ${_{1}\mathbf {c}}$ for the linear term, respectively.

Note that as we derive a solution for Eq. (27) by means of a minimization, the constraints from Eq. (21) and Eq. (23) are only satisfied to a certain extent, thus yielding a remaining error in terminal velocity and position, see Sect. 6. The same applies to the rotational constraint in Eq. (19). For mean values and standard deviations, derived for the conducted experiments, of ${_{\omega}\mathbf {c}}$, ${_{0}\mathbf {c}}$, and ${_{1}\mathbf {c}}$, see Appendix B.

### Nelder–Mead algorithm

In this work, optimizations are performed by means of the fmin function from Scipy (version 1.2.1), a package for scientific computing in Python [40]. The fmin function is an implementation of the Nelder–Mead algorithm [39] with the purpose of minimizing an objective function

29 $$ z = f(x) $$

by variation of $x$. The minimization is initialized by an initial guess for $x$ denoted as $x_{0}$. The minimization terminates if the absolute difference of two consecutive parameters $x_{\mathrm{j-1}}$ and $x_{\mathrm{j}}$ is less than or equal to a user-defined tolerance $x_{\mathrm{tol}}$

30 $$ \Vert x_{\mathrm{j}} - x_{\mathrm{j-1}} \Vert _{1} \le x_{ \mathrm{tol}}, $$

and if consecutive objective function values $z_{\mathrm{j-1}}$ and $z_{\mathrm{j}}$ meet the convergence criterion

31 $$ \Vert z_{\mathrm{j}} - z_{\mathrm{j-1}} \Vert _{1} \le f_{ \mathrm{tol}}. $$

For the following, we define the tolerances

32 $$ x_{\mathrm{tol}} = f_{\mathrm{tol}} = 1 \times 10^{-9}. $$

If the minimization terminates, the evaluated parameters $x$ that led to the smallest value of $z$ are denoted as optimal. However, the latter parameters could be local minima if the global minimum was not found.

### Correction of angular velocity

As orientation is required to compute translational velocity and position, see Fig. 2, it is thus required to optimize rotations first. To derive a solution for ${_{\omega}\mathbf {c}}$, see Eq. (20), a minimization of the objective function $f({_{\omega}\mathbf {c}})$,

33 $$ \min _{{{_{\omega}\mathbf {c}}} \ \in \ \mathbb{R}^{3}}\ f({_{\omega}\mathbf {c}})= {\Vert \Delta \pmb{\theta}({_{\omega}\mathbf {c}}) \Vert _{1} }, $$

is performed, where $\Delta \pmb{\theta}$ are angles describing the error in the terminal orientation ${\mathbf {R}_{n}}$, derived by means of the matrix logarithm. To initialize the minimization, the start values for ${_{\omega}\mathbf {c}}$ are defined as

34 $$ {_{\omega}\mathbf {c}}_{0} = \begin{bmatrix} 0 & 0 & 0 \end{bmatrix} ^{\mathrm{T}}. $$

If minimization from Eq. (33) terminates, as Eq. (30) and Eq. (31) are satisfied, optimal coefficients ${_{\omega}\mathbf {c}}$ are obtained. Therefore, the optimized angular velocity ${^{\textrm {S}_{i}}{\pmb{\omega}}_{\mathrm{opt}}}$ from Eq. (20) is utilized to compute the optimized incremental rotation vector ${}^{\textrm {S}}\pmb{\Omega}_{\mathrm{opt}}$ following Eq. (6). Further, the latter rotation vector is applied to Eq. (3), yielding optimized rotations

35 $$ \mathbf{R}_{\mathrm{opt},i+1}= \mathbf{R}_{\mathrm{opt},i} \exp (^{ \textrm {S}}\tilde{\pmb{\Omega}}_{\mathrm{opt}}). $$

Note that Eq. (33) can have multiple solutions that satisfy the constraint from Eq. (19). Consider an experiment with a duration of 2 s and a rotation about a single axis. Then, an angular velocity correction of ${_{\omega}\mathbf {c}}= \pi~\text{rad}\,\text{s}^{-1}$ yields the same terminal orientation as ${_{\omega}\mathbf {c}}= 0~\text{rad}\,\text{s}^{-1}$. Thus, we require that

36 $$ \Vert {_{\omega}\mathbf {c}}\Vert _{1} < 0.5\ \frac{2 \pi}{t_{\mathrm{n}}}, $$

as the expected deviation of rotations is considerably smaller than one full revolution. Note that the minimization algorithm provides the option to include a second term in Eq. (33) in future work, covering orientation derived from magnetometer and accelerometer investigations [24].

### Correction of translational acceleration

To compute optimal accelerations, see Eq. (28), we seek a solution of the correction coefficients ${_{0}\mathbf {c}}$ and ${_{1}\mathbf {c}}$, which are derived by minimization of the objective function $f({_{\mathrm{a}}\mathbf {c}})$,

37 $$ \min _{{_{0}\mathbf {c}}, {_{1}\mathbf {c}}\ \in \ \mathbb{R}^{3}}\ f({_{\mathrm{a}}\mathbf {c}})=\Vert \Delta\mathbf{v}({_{\mathrm{a}}\mathbf {c}}) \Vert _{2} ^{2}\ w_{\mathrm{v}} + \Vert \Delta\mathbf{p}({_{\mathrm{a}}\mathbf {c}}) \Vert _{2} ^{2}\ w_{ \mathrm{p}}, $$

where $\Delta \mathbf{v}$ describes the error of velocity and $\Delta \mathbf{p}$ is the error of the computed terminal position compared to the reference position, see Eq. (22) and Eq. (24), respectively. In Eq. (37), $w_{\mathrm{v}}$ and $w_{\mathrm{p}}$ denote weights that can be adjusted to compensate for different magnitude orders of $\Delta \mathbf{v}$ and $\Delta \mathbf{p}$, respectively. However, in this paper, the weights are equal,

38 $$ w_{\mathrm{v}} = w_{\mathrm{p}}, $$

as the orders of magnitudes are in the same range, see Appendix A. To initialize the minimization from Eq. (37), the start values for ${_{0}\mathbf {c}}$ and ${_{1}\mathbf {c}}$ are defined as

39 $$ {_{0}\mathbf {c}}_{0} = \begin{bmatrix} 0 & 0 & 0 \end{bmatrix} ^{\mathrm{T}} , \ {_{1}\mathbf {c}}_{0} = \begin{bmatrix} 0 & 0 & 0 \end{bmatrix} ^{\mathrm{T}}. $$

**Fig. 3:**
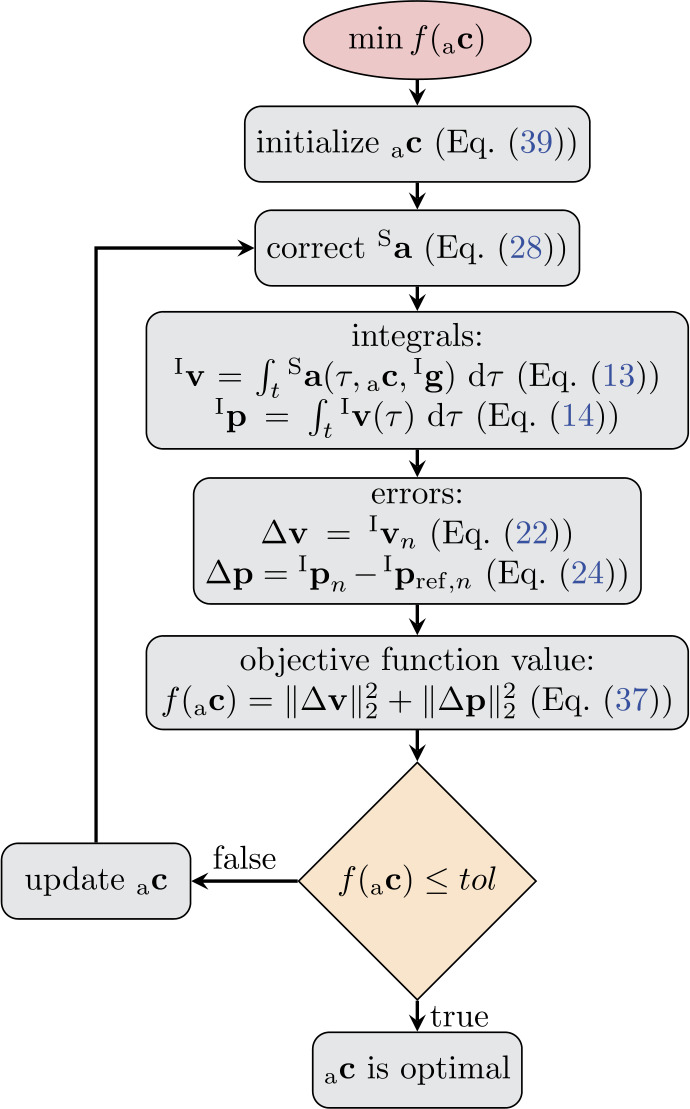
Flowchart of the optimization algorithm with the purpose of minimizing velocity and position errors by variation of correction coefficients on acceleration level (Color figure online)

If the minimization from Eq. (37) terminates, optimal coefficients ${_{0}\mathbf {c}}$, ${_{1}\mathbf {c}}$ are derived. Thus, the optimized translational velocity and position can be derived by computation of Eq. (13) and Eq. (14), respectively. However, optimized accelerations ${^{\textrm {S}}{\mathbf{a}}_{\mathrm{opt}}}$ from Eq. (28) instead of measured acceleration ${^{\textrm {S}}\mathbf{a}}$ are used. In addition, a substitution of $\mathbf{R}$ by $\mathbf{R}_{\mathrm{opt}}$ from Eq. (35) is performed, yielding

40 $$ ^{\textrm {I}}\mathbf{v}_{\mathrm{opt}}(t)=\int _{0}^{t} \left ( \mathbf{R}_{\mathrm{opt}}(\tau ) \ {^{\textrm {S}}\mathbf{a}_{ \mathrm{opt}}}(\tau ,{_{\mathrm{a}}\mathbf {c}}) - {^{\textrm {I}}\mathbf{g}} \right ) \mathrm{d}\tau , $$

41 $$ ^{\textrm {I}}\mathbf{p}_{\mathrm{opt}}(t)= \int _{0}^{t} \left ( \int _{0}^{ \tau _{2}} \left ( \mathbf{R}_{\mathrm{opt}}(\tau _{1}) \ {^{\textrm {S}} \mathbf{a}_{\mathrm{opt}}}(\tau _{1} ,{_{\mathrm{a}}\mathbf {c}}) - {^{\textrm {I}} \mathbf{g}} \right ) \mathrm{d}\tau _{1}\right ) \mathrm{d}\tau _{2}, $$

for optimized velocity and position, respectively. A flowchart of the optimization is shown in Fig. 3, covering the major steps.

#### Simplified correction without rotations

The purpose of simplification, such that the EOM rely solely on translational terms, is twofold. First, the coefficients for the polynomial correction of accelerations can be derived analytically. This further allows verification of the present optimization method from Sect. 3.4 through comparison of solutions derived by the latter method with the analytical solutions. Secondly, investigating the influence of rotations by comparing solutions for simultaneous translation and rotation with solutions for simplified motion is possible, see Sect. 6.

Simplification of the general nonlinear EOM can be derived if the investigated system has constant orientation. This leads to a linear EOM, as nonlinearity is caused by rotations. Considering constant orientation, it follows that

42 $$ \mathbf{R}_{i} = \mathbf{I}_{3}, \ \forall i \in \{0,1,2, \ldots ,n\}, $$

where $\mathbf{R}_{i}$ is the rotation matrix that performs a transformation from sensor frame (S) to initial frame (I) and $\mathbf{I}_{3} \in \mathbb{R}^{3\times 3}$ denotes the identity matrix. Thus, if orientation is constant over time, the sensor frame (S) corresponds to the initial frame (I). Hence, the left upper superscript is dropped in this section. By means of Eq. (42), the velocity from Eq. (40) and the position from Eq. (41) take the simplified form of

43 $$ \mathbf{v}_{\mathrm{opt}}(t)=\int _{t} \left ( {{\mathbf{a}}(\tau )} + {_{0}\mathbf {c}}+ {_{1}\mathbf {c}}\ \tau - {\mathbf{g}} \right ) \mathrm{d}\tau , $$

44 $$ \mathbf{p}_{\mathrm{opt}}(t)= \int _{t} \left ( \int _{\tau _{2}} \left ( {{\mathbf{a}}(\tau _{1})} + {_{0}\mathbf {c}}+ {_{1}\mathbf {c}}\ \tau _{1} - { \mathbf{g}}\right ) \mathrm{d}\tau _{1}\right ) \mathrm{d}\tau _{2}, $$

respectively. Considering Eq. (22), and comparing Eq. (13) with Eq. (43) derives

45 $$\begin{aligned} \mathbf{v}_{\mathrm{opt},n} &= {\mathbf {v}_{n}}- \Delta \mathbf{v} \\ &=\int _{0}^{t_{\mathrm{n}}}\left ( {{\mathbf{a}}(\tau )- { \mathbf{g}}}\right ) \mathrm{d}\tau + \int _{0}^{t_{\mathrm{n}}} \left ( {{_{0}\mathbf {c}}+ {_{1}\mathbf {c}}\ \tau } \right ) \mathrm{d}\tau . \end{aligned}$$

Therefore,

46 $$\begin{aligned} -\Delta \mathbf{v} &= {_{0}\mathbf {c}}\ t_{n} + {_{1}\mathbf {c}}\ \frac{t_{n}^{2}}{2} \\ &= {_{0}\mathbf {c}}\ k_{0} + {_{1}\mathbf {c}}\ k_{1}. \end{aligned}$$

Analogous to Eqs. (45)-(46) we derive an analytical solution for $\Delta \mathbf{p}$ by comparison of Eq. (14) with Eq. (44) under consideration of Eq. (24) yielding

47 $$\begin{aligned} -\Delta \mathbf{p}&= {_{0}\mathbf {c}}\ \frac{t_{n}^{2}}{2} + {_{1}\mathbf {c}}\ \frac{t_{n}^{3}}{6} \\ &= {_{0}\mathbf {c}}\ k_{2} + {_{1}\mathbf {c}}\ k_{3}. \end{aligned}$$

Rearranging Eq. (46) and Eq. (47) derives the system of equations in matrix form for computation of the polynomial coefficients,

48 $$ \begin{bmatrix} {_{0}\mathbf {c}}^{\mathrm{T}} \\ {_{1}\mathbf {c}}^{\mathrm{T}} \end{bmatrix} = \frac{1}{k_{0} k_{3} - k_{1} k_{2}} \begin{bmatrix} k_{3}&-k_{1} \\ -k_{2}&k_{0} \end{bmatrix} \begin{bmatrix} -\Delta\mathbf{v}^{\mathrm{T}} \\ -\Delta\mathbf{p}^{\mathrm{T}} \end{bmatrix} . $$

## Calibration

Calibration of low-cost IMUs is crucial, as uncalibrated IMU data can hardly be further processed to yield consistent orientation and position [12]. Therefore, each utilized IMU is subject to gyrometer and accelerometer calibration. The objective of the following calibrations is to derive parameters that relate measured quantities to ideal (reference) quantities. These parameters, in the form of matrices and vectors, can then be applied to raw IMU data from any experiment to derive calibrated data. However, parameters for accelerometer and gyrometer calibration are different and are derived from different experiments.

### Error model

This section deals with error modeling of IMUs, specifically 3-axis accelerometers and gyrometers. Most of these sensor errors can be classified as scaling $\mathbf{S}$, nonorthogonality $\mathbf{N}$, misalignment $\mathbf{M}$, and bias $\mathbf{b}$, which are of a deterministic kind. A detailed description of the latter deterministic errors can be found in [44]. Additionally, there is the stochastic noise term $\mathbf{r}$, which we, however, do not consider in the present calibration. Since some definitions are identical for the different sensors, left-hand indices are introduced, denoting accelerations ($\mathrm{a}$) and angular velocities ($\omega $).

The error models for measured translational accelerations $\mathbf{ \bar{a}} \in \mathbb{R}^{3\times n}$ and measured angular velocities $\bar{\pmb{\omega}} \in \mathbb{R}^{3\times n}$ are defined for one time step $i \in \{0,1,2,\ldots,n\}$ as [45]

49 $$\begin{aligned} \mathbf{ \bar{a}}_{i} &= {_{\mathrm{a}}\mathbf{S}}\ {_{\mathrm{a}} \mathbf{N}}\ {_{\mathrm{a}}\mathbf{M}}\ \mathbf{a}_{i} + {_{ \mathrm{a}}\mathbf{b}} + {_{\mathrm{a}}\mathbf{r}}, \end{aligned}$$

50 $$\begin{aligned} \bar{\pmb{\omega }}_{i} &= {{_{\omega }\mathbf{S}}}\ {_{\omega } \mathbf{N}}\ {_{\omega }\mathbf{M}}\ \pmb{\omega }_{i} + {_{\omega } \mathbf{b}} + {_{\omega }\mathbf{r}}, \end{aligned}$$

where $\mathbf{a}$ and $\pmb{\omega}$ are calibrated translational accelerations and calibrated angular velocities, respectively. As calibrated values are of interest for application and noise is not considered we define the calibration matrix

51 $$ \mathbf{C}=\left ( {\mathbf{S}} {\mathbf{N}} {\mathbf{M}}\right )^{-1}= {\mathbf{M}^{-1}} {\mathbf{N}^{-1}} {\mathbf{S}^{-1}}, $$

then we drop the noise terms ${_{\mathrm{a}}\mathbf{r}}$ and ${_{\omega}\mathbf{r}}$, and rearrange Eqs. (49)-(50), yielding

52 $$\begin{aligned} \mathbf{a}_{i} &= {_{\mathrm{a}} \mathbf{C}} (\mathbf{ \bar{a}}_{i} - {_{ \mathrm{a}}\mathbf{b}}), \end{aligned}$$

53 $$\begin{aligned} {\pmb{\omega }}_{i} &= {_{\omega }\mathbf{C}} (\bar{\pmb{\omega }}_{i} - {_{\omega }\mathbf{b}} ), \ \forall i \in \{0,1,2, \ldots , n\}. \end{aligned}$$

Note that in the proposed calibration, we compute the calibration matrix $\mathbf{C}$. If the individual scaling $\mathbf{S}$, nonorthogonality $\mathbf{N}$, and misalignment $\mathbf{M}$ terms are of particular interest, the reader may consider [45] where the Cholesky- and LU-decomposition are used to derive the individual components.

### Angle-domain gyrometer calibration

The angle-domain calibration [45, 46] relies on a comparison of computed angles with reference angles. The reference angles are derived from a calibration measurement sequence containing three successive rotations about the reference axes, which correspond to the robot-effector coordinate system.

**Fig. 4:**
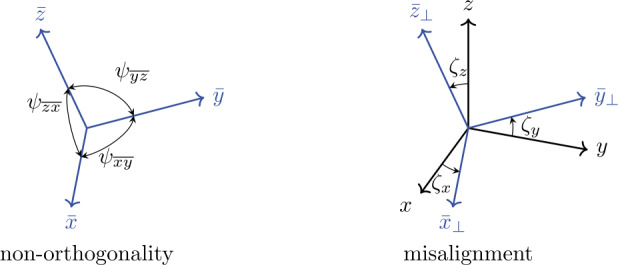
Reference coordinate system ($x,y,z$) denoted as ℱ and wrongly scaled, nonorthogonal, and misaligned coordinate system of the sensor ($\bar{x},\bar{y},\bar{z}$) denoted as $\overline {\mathcal {F}}$. Angles $\psi $ describe nonorthogonality. Angles $\zeta $ describe misalignment between an already orthogonalized sensor coordinate system ($\bar{x}_{\bot}$, $\bar{y}_{\bot}$, $\bar{z}_{\bot}$) and ℱ(Color figure online)

In the context of calibration, we define two different coordinate systems. The coordinate system _*ω*_$\overline {\mathcal {F}}$ is related to the wrongly scaled, nonorthogonal, and misaligned coordinate triad ($\bar{x},\bar{y}, \bar{z}$). Moreover, there is the coordinate system ℱ  that is related to the reference triad ($x,y,z$), see Fig. 4. For elimination of the latter errors, we seek a solution of the calibration matrix ${_{\omega }\mathbf{C}}$, which corrects computed angles regarding scaling, nonorthogonality and misalignment such that

54 $$ \boldsymbol{\Phi} = {_{\omega }\mathbf{C}} \ \bar{\boldsymbol{\Phi}}, $$

where

55 $$ {\boldsymbol{\Phi}} = \begin{bmatrix} _{{1}}{\pmb{\phi}} & _{{2}}{\pmb{\phi}} & _{{3}}{\pmb{\phi}} \end{bmatrix} $$

contains predefined reference rotation vectors for three single measurements in columns. The linear independent rotation vectors of ${\boldsymbol{\Phi}}$ are defined as

56 $$ _{{1}}{\pmb{\phi}} = { \begin{bmatrix} \alpha & 0 & 0 \end{bmatrix} }^{\mathrm{T}}\ , \ _{{2}}{\pmb{\phi}} = { \begin{bmatrix} 0 & \beta & 0 \end{bmatrix} }^{\mathrm{T}}\ , \ _{{3}}{\pmb{\phi}} = { \begin{bmatrix} 0 & 0 & \gamma \end{bmatrix} }^{\mathrm{T}}, $$

where $\alpha $, $\beta $, and $\gamma $ define the angle of rotation about the $x$-, $y$-, and $z$-axis, respectively. Hence, we rotate the IMU about the $x$-axis by the angle $\alpha $ in the first measurement, rotate about the $y$-axis by the angle $\beta $ in the second measurement, and rotate about the $z$-axis by the angle $\gamma $ in the third measurement. The matrix

57 $$ \bar{\boldsymbol{\Phi}} = \begin{bmatrix} _{{1}}\bar{\pmb{\phi}} & _{{2}}\bar{\pmb{\phi}} & _{{3}} \bar{\pmb{\phi}} \end{bmatrix} $$

is computed from averaged, bias-corrected, measured local angular velocities from three individual measurements. Hence, each column of Eq. (57) results from the multiplication of the averaged angular velocity with the duration $T=n \Delta t$, similar to [9],

58 $$ \bar{\pmb{\phi}} =T \ \frac{1}{2(n-1)}\ \sum _{i=0}^{n-1}{ \left ( ( {^{ \textrm {S}_{i}}\bar{\pmb{\omega}}_{i}}-{_{\omega}\mathbf{b}}) +( {^{ \textrm {S}_{i}}\bar{\pmb{\omega}}_{i+1}}-{_{\omega}\mathbf{b}} ) \right ) }, $$

utilizing the trapezoidal rule for averaging, in order to be consistent with the time integration, see Eq. (6). Thus, $\bar{\boldsymbol{\Phi}}$ represents rotations in _*ω*_$\overline {\mathcal {F}}$ while rotating about the axes of ℱ. Under the assumption of small errors in the angular velocities, we assume that $\bar{\boldsymbol{\Phi}}$ is regular. Therefore, rearranging Eq. (54) gives the calibration matrix

59 $$ {_{\omega }\mathbf{C}} = \boldsymbol{\Phi} \ \bar{\boldsymbol{\Phi}}^{-1}. $$

This calibration matrix can directly be applied to the measured angular velocities, see Eq. (53). The bias $_{\omega}\mathbf{b}$ corresponds to the average angular velocity at standstill

60 $$ _{\omega}\mathbf{b} = \frac{1}{j}\sum _{j}{\bar{\pmb{\omega}}_{j}}, $$

and is determined using $j$ samples. Within this paper, the bias is determined at the beginning of a measurement considering 400 samples (equals 1 second). In addition, the bias is not only determined in the calibration measurements, but also in every other experiment, which can easily be achieved by persisting at standstill for at least 1 second at the beginning of a measurement. Note that nonorthogonality and misalignment errors are time invariant, however, scaling and bias errors are time and temperature dependent [45]. Thus, the best motion-reconstruction results are obtained by calibrating directly before an experiment.

### Accelerometer calibration

The accelerometer coordinate system _a_$\overline {\mathcal {F}}$ is also subject to wrong scaling, nonorthogonality, misalignment, and bias. Thus, we utilize $k$ samples of measured accelerations $\bar{\mathbf{a}}$ from a standstill IMU at different orientations and corresponding reference accelerations ${\mathbf{a}}$ to derive a calibration matrix ${_{\mathrm{a}} \mathbf{C}}$ and bias ${_{\mathrm{a}}\mathbf{b}}$. For acquisition of IMU accelerations and reference accelerations, see Sect. 5.4.1. To derive a solution for calibration matrix ${_{\mathrm{a}} \mathbf{C}}$ and bias ${_{\mathrm{a}}\mathbf{b}}$ we solve a least-squares minimization problem. Thus, we rearrange Eq. (52) into the steady-state form yielding

61 $$ \mathbf{y} = \mathbf{P}\,\mathbf{x}, $$

with

62 $$\begin{aligned} \mathbf{y} &= \mathbf{a}\ ,\ \mathbf{y}\in \mathbb{R}^{3\times k} \end{aligned}$$

63 $$\begin{aligned} \mathbf{P} &= \begin{bmatrix} {_{\mathrm{a}} \mathbf{C}} & {_{\mathrm{a}} \mathbf{C}} {_{\mathrm{a}} \mathbf{b}} \end{bmatrix}\ ,\ \mathbf{P}\in \mathbb{R}^{3\times 4} \end{aligned}$$

64 $$\begin{aligned} \mathbf{x} &= \begin{bmatrix} \bar{\mathbf{a}} \\ -1 \end{bmatrix}\ ,\ \mathbf{x}\in \mathbb{R}^{4\times k}. \end{aligned}$$

In Eqs. (61)-(64), $\mathbf{y}$ are the calibrated accelerations, $\mathbf{P}$ defines a parameter matrix including all calibration parameters, and $\mathbf{x}$ is composed of measured accelerations, and $(-1)$ for subtraction of bias from measured accelerations. Equation (61) is an overdetermined system of equations with $k$ equations and 12 unknown parameters. Thus, a solution for the parameter matrix $\mathbf{P}$ is derived by

65 $$ \mathbf{P}= \mathbf{y}\,\mathbf{x}^{+}, $$

utilizing the Moore–Penrose generalized inverse [47] $\mathbf{x}^{+} = {\mathbf{x}^{\mathrm{T}}}(\mathbf{x}\,{\mathbf{x}^{ \mathrm{T}}})^{-1} \in \mathbb{R}^{k\times 4}$ as $\mathbf{P} \in \mathbb{R}^{3\times 4}$ is not a square matrix. Note that (65) is a minimal norm solution to the Least-Squares minimization problem [48]

66 $$ \mathrm{min}_{\mathrm{(P)}}\Vert \mathbf{P}\mathbf{x}-\mathbf{y} \Vert ^{2} _{2} . $$

## Measurement-data acquisition

The presented algorithms are developed with the overall objective of motion reconstruction of particles in snow avalanches, where the start- and end-orientation can be computed using magnetometer and accelerometer data [16]. Additionally, the position can be determined utilizing a GNSS. However, these orientations and positions are not valid for validation of computed trajectories as there is no reference. Thus, we use an industrial manipulator to move along predefined trajectories, where reference orientations and positions are provided by the robot controller. As compared to [8–10, 12], calibration and experiments are performed on a manipulator without changing the clamping of the IMU in between. Hence, each IMU is calibrated with respect to the robot coordinate system, which is further used to represent the reference trajectories and evaluate the deviation of computed trajectories. Additionally, each experiment is performed five times, utilizing five individual IMUs ($S_{1}$–$S_{5}$) of the same type successively. To minimize orientation and position errors due to mounting, we utilize a special flange for backlash-free clamping of the IMUs. Once an IMU is clamped, we perform two experiments for calibration followed by one experiment with motion at constant orientation and one experiment with simultaneous translation and rotation. In each experiment, we measure an additional two seconds prior and subsequent to motion, thus at standstill. This standstill data is used for sensor-bias determination and is cut out thereafter.

### Measurement system setup

Measurement data from the following experiments were acquired by a measuring system called AvaNode [16].

#### Measuring system (AvaNode)

The AvaNode was initially built to capture the inflow dynamics of snow avalanches and provides functions that are neither used for nor affect the presented experiments, e.g., retrieval systems, GNSS, and magnetometer. However, we utilized the main component of the latter system; the IMU of type MPU9250.^4^ This IMU is mounted in a 3D printed cube with 100 mm edge length, such that the IMU coordinate system coincides with the geometric center of the cube. There is also a robust housing for infield measurements, which is, however, not used in the present paper. Data provided by the IMU is stored on a SD-Card by means of an Adafruit Feather m0 microcontroller with a sampling rate of 400 Hz.

#### Manipulator

**Fig. 5:**
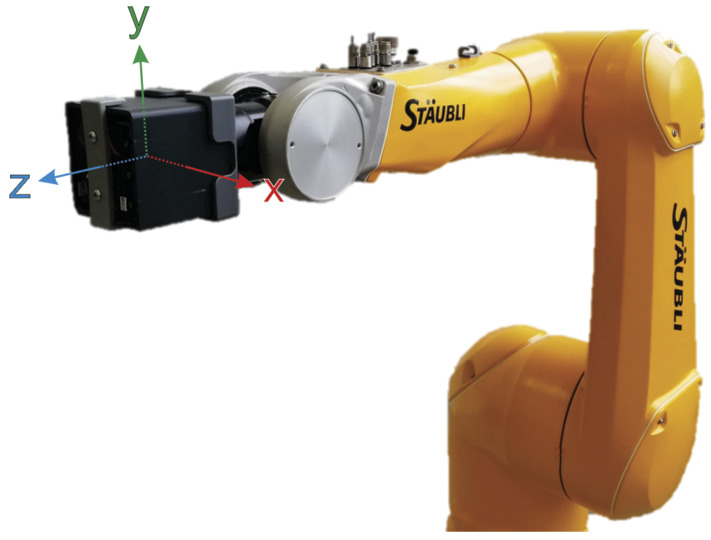
Measurement system setup with a 6R manipulator to perform motion and an IMU on the effector to measure motion. Additionally, the coordinate system of the robot effector corresponds to the coordinate system of the calibrated IMU accelerometer and gyrometer (Color figure online)

To perform multiple experiments with individual IMUs with the same motion, we utilized an industrial manipulator with 6 DOF of type Stäubli TX2-90L, see Fig. 5. This manipulator has a repetitive positioning accuracy of ±0.035 mm, a reach of 1200 mm, and is able to move objects ≤6 kg. In addition, the robot controller provides reference positions and orientations during motion. However, this reference data is provided with a sampling frequency of 250 Hz. Thus, we resampled measured reference data to 400 Hz.

### Translational motion at constant orientation

**Fig. 6:**
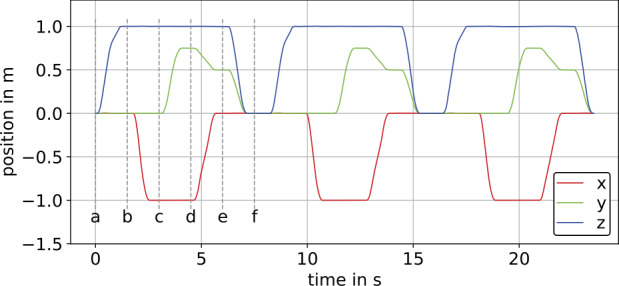
Reference positions with respect to the initial frame (I), provided by the Stäubli robot controller, for the experiment at constant orientation and the experiment with simultaneous translation and rotation (Color figure online)

**Fig. 7:**
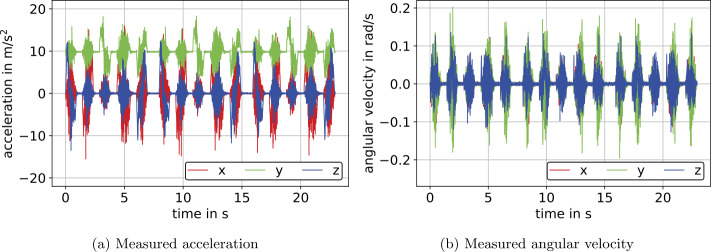
Calibrated measurement data for the experiment at constant orientation on the example of $S_{2}$ (Color figure online)

The experiment with translational motion at constant orientation comprises three identical trajectories performed in succession with a standstill period of 1 s in between, as shown in Fig. 6. In addition, each of these three trajectories consists of three linear translations in the direction of one sensor axis at a time and two subsequent planar linear translations thereafter, see Fig. 6. In between the individual translations there is a standstill period of 0.5 s. The time stamps and position values corresponding to standstill periods denoted as (a, b, c, d, e, f) are shown in Fig. 6 and Table 1, respectively. The experiments were conducted by moving the manipulator at maximum joint speed resulting in peak values of 2 m s^−1^ for the $z$-axis and 1.5 m s^−1^ for the $x$- and $y$-axes velocities. Further, the manipulator accelerated the IMU with the acceleration peaking at ≈10 m s^−2^ according to the measured accelerations shown in Fig. 7a, where gravity effects the $y$-axis. The errors in measured angular velocities are caused by translational acceleration due to sensor crosscoupling and are shown in Fig. 7b.

**Table 1:** Positions with respect to the initial frame (I) in meters at standstill periods of the experiment at constant orientation. The markers denote time stamps of the standstill periods corresponding to Fig. 6

marker	a	b	c	d	e	f
*x*-axis	0	0	−1	−1	0	0
*y*-axis	0	0	0	0.75	0.5	0
*z*-axis	0	1	1	1	1	0

### Simultaneous translation and rotation

**Fig. 8:**
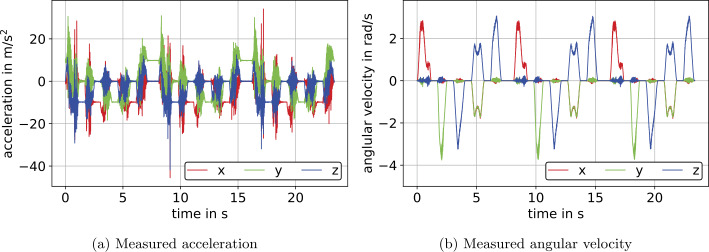
Calibrated measurement data for the experiment with simultaneous translation and rotation on the example of IMU $S_{2}$ (Color figure online)

In the present section, the translational part is equivalent to the motion described in Sect. 5.2, however, with simultaneous rotations. The IMU is rotated about its axes by an angle according to Table 2. Rotation is performed such that rotational increments are evenly distributed over the course of translation. In Fig. 8, measured accelerations and angular velocities for the measurement with simultaneous translation and rotation are displayed. As in Sect. 5.2, gravity in Fig. 8a effects the $y$-axis of the initial frame and is eliminated following Eq. (12). In Fig. 8b, we see the correspondence of angular velocities to the rotations denoted in Table 2.

**Table 2:** Rotations with respect to the sensor frame (S) in radians between standstill periods of the experiment with simultaneous translation and rotation. The markers denote time stamps of the standstill periods corresponding to Fig. 6

marker	a–b	b–c	c–d	d–e	e–f
*x*-axis	*π*/2	0	0	-*π*/2	0
*y*-axis	0	-*π*/2	0	-*π*/2	0
*z*-axis	0	0	-*π*/2	*π*/2	*π*/2

### Calibration measurements

To calibrate the accelerometer and gyrometer, applying the methods from Sect. 4, it is required to perform specific measurements for calibration data acquisition.

#### Accelerometer measurements for six-position calibration

**Fig. 9:**
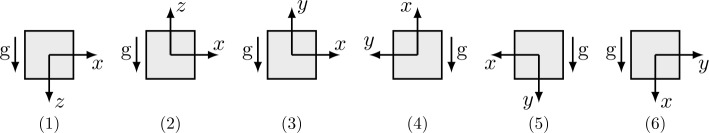
Individual orientations of the six-position calibration in chronological sequence as they were conducted in this paper. All orientations are shown with respect to a common viewpoint

In this section, we present the measurements performed with the purpose of accelerometer calibration. Here, an IMU was placed in six different orientations utilizing the manipulator, such that each of the three sensor axes is successively parallel to the gravity vector and has an equal direction for the first three measurements and the opposite direction for the remainder. The sequence of the six orientations as we used it in this paper is shown in Fig. 9. In each orientation, the IMU was held for 5 s to measure accelerations at standstill, corresponding to reference accelerations

67 $$ _{1}\mathbf{a}= \begin{bmatrix} 0 \\ 0 \\ -g \end{bmatrix} ,\, _{2}\mathbf{a}= \begin{bmatrix} 0 \\ 0 \\ g \end{bmatrix} ,\, _{3}\mathbf{a}= \begin{bmatrix} 0 \\ g \\ 0 \end{bmatrix} ,\, _{4}\mathbf{a}= \begin{bmatrix} g \\ 0 \\ 0 \end{bmatrix} ,\, _{5}\mathbf{a}= \begin{bmatrix} 0 \\ -g \\ 0 \end{bmatrix} ,\, _{6}\mathbf{a}= \begin{bmatrix} -g \\ 0 \\ 0 \end{bmatrix} , $$

for orientations (1) to (6) according to Fig. 9. Note that in Eq. (67), accelerations for one time step are denoted. Further, note that due to their functionality, an axis of an IMU measures positive gravitation when this sensor axis is parallel, but points in the opposite direction to the gravitation vector. Apparently, the raw measurement data also comprises the motion from one orientation to another. Therefore, the latter motions are cut from the measurement data. The remaining data, which are measured accelerations at standstill, are arranged into a sequence resulting in an acceleration vector for calibration

68 $$ \bar{\mathbf{a}}= \begin{bmatrix} _{1}\bar{\mathbf{a}} & _{2}\bar{\mathbf{a}} & _{3}\bar{\mathbf{a}} & _{4} \bar{\mathbf{a}} & _{5}\bar{\mathbf{a}} & _{6}\bar{\mathbf{a}} \end{bmatrix} , $$

and corresponding reference accelerations

69 $$ \mathbf{a}= \begin{bmatrix} _{1}\mathbf{a} & _{2}\mathbf{a} & _{3}\mathbf{a} & _{4}\mathbf{a} & _{5} \mathbf{a} & _{6}\mathbf{a} \end{bmatrix} . $$

The acquired acceleration vectors $\bar{\mathbf{a}}$ and $\mathbf{a}$ can then be utilized to compute calibration parameters, see Sect. 4.3.

#### Gyrometer measurements for calibration

The angle-domain calibration denotes comparison of computed angles with reference angles to derive calibration parameters. Computed angles result from time integration of the measured angular velocities and the reference angles are provided by the robot controller. For this purpose, we performed measurements containing successive rotations about the reference frame axes. In general, the rotation angles about $x$-, $y$-, and $z$-axes are defined as $\alpha $, $\beta $, and $\gamma $, respectively. In the present gyrometer calibration measurements, we chose

70 $$ \alpha = \beta = \gamma = \pi\ \text{rad}. $$

Therefore, Eq. (55) takes the special form of

71 $$ {\boldsymbol{\Phi}} = \pi \begin{bmatrix} 1 & 0 & 0 \\ 0 & 1 & 0 \\ 0 & 0 & 1 \end{bmatrix} . $$

**Fig. 10:**
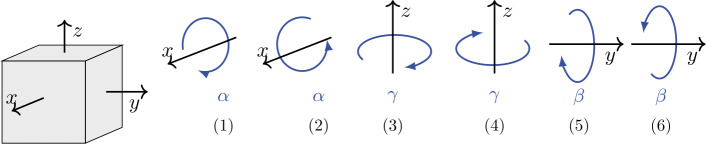
Successive rotations about one axis of the gyrometer for calibration purpose (Color figure online)

Rotations by means of the manipulator are performed such that each rotation is followed by an opposite rotation, back to the initial orientation, see Fig. 10. In addition, there is one common pivot for all rotations that coincides with the center of the reference coordinate system ℱ. This results in a measurement comprising solely rotations without translations. In between the individual rotations, which are performed in the sequence shown in Fig. 10, there is a standstill period of 2 s, see Fig. 15 displaying the example of IMU $S_{2}$.

Note that there is also the approach to calibrate in the angular velocity domain [12]. However, this requires that the sensor is rotated with constant angular velocity. Apparently, we could realize this approach by means of the manipulator. However, the presented approach does not require special equipment and is therefore preferred.

## Experimental results

The methods for motion reconstruction and optimization, satisfying constraints on velocity, position, and angular velocity by means of polynomial corrections on measured quantities, were applied to experimental measurement data. Specifically, two different experiments (motion with and without rotation) were performed multiple times by means of an industrial robot. Data was successively acquired by five individual IMUs of the same type. Each of the IMUs underwent calibration. Additionally, reference positions and orientations were provided by the robot controller.

The presented calibration, motion reconstruction, and optimization methods were computed in Python (version 3.7) and were utilized to derive the results in the following. Input-file and data-array handling, computation of mean values and norms was performed by means of Numpy (version 1.16.4) [49]. Reference data resampling and the Nelder–Mead optimization algorithm were utilized from Scipy (version 1.2.1) [40]. In addition, parts of Sect. 2 regarding Lie groups were computed with the help of Exudyn (version 1.0.151) [50].

### Reconstructed motion without optimization

In this section, the motion-reconstruction algorithm from Sect. 2 was applied to calibrated measurement data. The latter data was acquired from two different experiments. Specifically, one experiment with translations at constant orientation and another experiment with the same translations, however, with simultaneous rotations, see Fig. 7 and Fig. 8, respectively. Each of the two experiments was performed five times, using five successively mounted IMUs of the same type, denoted as $S_{1}$–$S_{5}$. For every IMU, the experiment with rotations was conducted first, followed by the experiment without rotations.

**Fig. 11:**
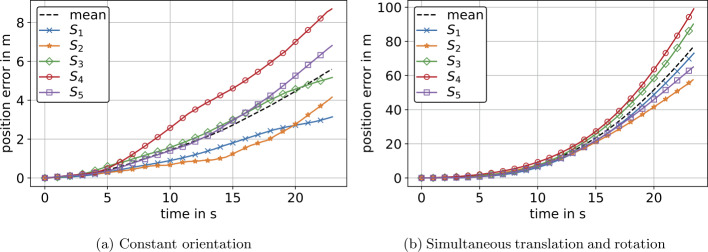
Euclidean norm of position errors $\mathbf{p}_{\mathrm{err}}$ for experiments at constant orientation (a) and with simultaneous translation and rotation (b) prior to optimization. $S_{1}$–$S_{5}$ denote five individual IMUs that correspond for (a) and (b). Additionally, the mean value of position errors of all IMUs is displayed (Color figure online)

The differences between the computed positions and reference positions for both experiments are shown in Fig. 11. In Fig. 11 and the following, the position errors ${}_{s}\mathbf{p}_{\mathrm{err}}$ for IMU $S_{j}$ are represented by the Euclidean norm

72 $$ _{j}\mathrm{p}_{\mathrm{err},i} = \Vert _{j}\mathbf{p}_{i} - \mathbf{p}_{\mathrm{ref},i} \Vert _{2},\ \forall i \in \{0,1,2, \ldots , n\}\ \mathrm{and} \ \forall j \in \{1,2, \ldots , 5\}, $$

where $\mathbf{p}$ is a vector of spatial positions derived from Eq. (14) and $\mathbf{p}_{\mathrm{ref}}$ are corresponding reference positions provided by the robot controller. Figure 11 and the following error plots also include a mean value of position errors derived by

73 $$ \hat{\mathrm{p}}_{\mathrm{err},i} = \frac{1}{5}\sum _{j=1}^{5}{_{j} \mathrm{p}_{\mathrm{err},i},\ \forall i \in \{0,1,2, \ldots , n\}}. $$

**Fig. 12:**
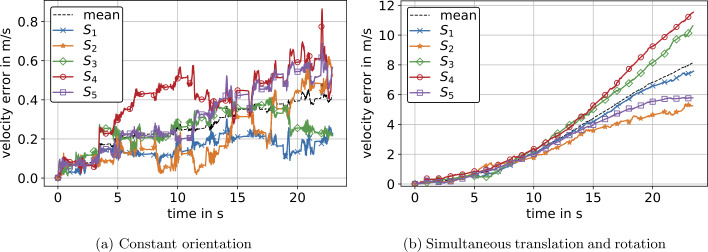
Euclidean norm of velocity errors $\mathbf{v}_{\mathrm{err}}$ from IMU $S_{1}$–$S_{5}$ for experiments at constant orientation (a) and with simultaneous translation and rotation (b) prior to optimization. Additionally, the mean value of velocity errors of all IMUs is displayed (Color figure online)

The errors in velocity are derived analogous to Eq. (72) and Eq. (73), respectively, yielding ${}_{s}\mathbf{v}_{\mathrm{err}}$ and $\hat{\mathbf{v}}_{\mathrm{err}}$, see Fig. 12. Referring to the mean values of errors in Fig. 11a and Fig. 11b, we see an approx. linear and quadratic error increase, respectively. This yielded an average maximum error of 5.6 m after 23 s for the experiment at constant orientation, where the absolute maximum error was 8.7 m, see Fig. 11a. For the experiment with rotations, there was an average maximum error of 76.6 m after 23 s and a maximum error of 99 m for sensor $S_{4}$, see Fig. 11b. Thus, for an experiment with a duration of 23 s, we investigated an approximately 13 times higher maximum error for measurements with rotations compared to measurements at constant orientation. The error values for each individual sensor are shown in Table 3 and Table 5 for experiments at constant orientation and experiments with rotations, respectively.

**Table 3:** Maximum and average error in position prior and subsequent to optimization for the experiment at constant orientation for sensors $S_{1}$–$S_{5}$

value		unit	$S_{1}$	$S_{2}$	$S_{3}$	$S_{4}$	$S_{5}$
nonopt.	max($\mathrm{p}_{\mathrm{err}}$)	m	3.145	4.161	5.171	8.703	6.819
	average($\mathrm{p}_{\mathrm{err}}$)	m	1.315	1.202	2.228	3.410	2.330
optimized	max($\mathrm{p}_{\mathrm{err}}$)	m	0.340	0.388	0.609	0.995	0.615
	average($\mathrm{p}_{\mathrm{err}}$)	m	0.204	0.193	0.330	0.434	0.274

### Optimization of experiments at constant orientation

**Fig. 13:**
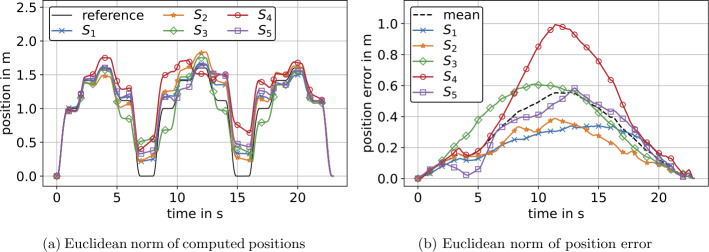
Computed position (a) and error of position (b) by means of optimized translational acceleration for the experiment at constant orientation. Here, (b) shows the norm of the deviation of computed positions to reference positions, not deviation of norms from (a) (Color figure online)

The present section deals with optimization of reconstructed velocities and positions derived from the experiment at constant orientation, see Fig. 11a. Specifically, as $\mathbf{R}=\mathbf{I}_{3}$ in this case, see Eq. (42), we applied the optimization from Sect. 3.4.1 to measured calibrated accelerations. In Fig. 13a, computed positions of all IMUs, as well as a reference position are shown. Comparing Fig. 13b to Fig. 11a, it can be seen that the terminal position error was eliminated by means of the present optimization. The deviation of the position constraint, see equation Eq. (23), is in the range of $10^{-9}$ and depends on the tolerance settings of the Nelder–Mead algorithm, see Sect. 3.2. The same applies to the deviation of the velocity constraint, see Eq. (21). For actual values of terminal velocity and position, we refer to Table 6 in Appendix A. In contrast to Sect. 6.1, the maximum error of optimized positions was approximately at measurement half-time, see Fig. 13b. Referring to the mean value in Fig. 13b, the error increased from zero to the maximum value in the first half of the measurement and decreased to zero in the second half. Additionally, the maximum of the mean error decreased from 5.6 m to 0.56 m, thus by approximately 90%, see Fig. 11a and Fig. 13b, respectively.

#### Validation of optimization with analytical solution

To validate the present Nelder–Mead optimization we compare the acceleration correction coefficients from Sect. 6.2 with their analytical solution, derived in Sect. 3.4.1. Table 4 shows mean values of the correction coefficients of sensor $S_{2}$, derived from both methods. In addition, Table 4 shows the errors of the correction coefficients, derived by means of the optimization, with respect to the analytical solution. These errors are in the range of $10^{-6}$ and thus validate the functionality of the optimization for the present work.

**Table 4:** Correction coefficients for sensor $S_{2}$ of the polynomial (${_{\mathrm{a}}\mathbf {c}}= {_{0}\mathbf {c}}+ {_{1}\mathbf {c}}\ t$) to satisfy velocity constraints derived analytically and with Nelder–Mead optimization. In addition, the error of the Nelder–Mead solution is compared to the analytical solution for sensor $S_{2}$

term	axis	analytical solution	optimization solution	error
_0_**c** (constant)	x	−7.52881 × 10^−3^	−7.53568 × 10^−3^	6.86658 × 10^−6^
	y	6.37839 × 10^−3^	6.37222 × 10^−3^	6.16618 × 10^−6^
	z	6.39584 × 10^−3^	6.39184 × 10^−3^	3.99919 × 10^−6^
_1_**c** (linear)	x	−8.80620 × 10^−4^	−8.80117 × 10^−4^	−5.03011 × 10^−7^
	y	−1.65037 × 10^−3^	−1.65001 × 10^−3^	−3.57965 × 10^−7^
	z	−1.21826 × 10^−3^	−1.21804 × 10^−3^	−2.16015 × 10^−7^

### Optimization of experiments with simultaneous translation and rotation

The optimization was also applied to IMU data acquired from experiments with simultaneous translation and rotation. Hence, angular velocity and acceleration were optimized to satisfy terminal orientation, velocity, and position constraints.

**Fig. 14:**
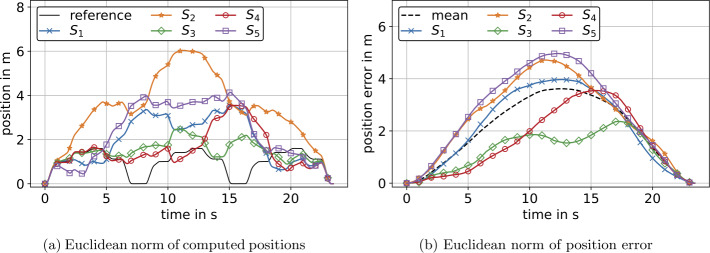
Computed position (a) and error of position (b) by means of optimized angular velocity and optimized translational acceleration for the experiment with simultaneous translation and rotation. Here, (b) shows the norm of deviation of computed positions to reference positions, not deviation of norms from (a) (Color figure online)

In Fig. 14a, the positions with respect to the start position for all sensors, as well as a reference position are shown. The remaining errors subsequent to optimization are displayed in Fig. 14b. In addition, Fig. 14b provides the mean value of remaining errors from sensors $S_{1}$–$S_{5}$. This mean error peaked at 3.6 m at approx. measurement half-time and thus decreased by approximately 95% compared to the maximum value of the mean errors of nonoptimized positions, see Fig. 11b. However, the worst performance of the optimization was investigated for sensors $S_{2}$ and $S_{5}$ with an error decrease of approximately 92%. The optimization yielded best results for sensor $S_{4}$, decreasing the maximum error from 99 m to 3.4 m and thus by approximately 96%. In addition, the average error over the measurement duration also decreased by approximately 93% with respect to mean values from all 5 sensors, see Table 5. As the position error is mainly driven by measurement duration, we assume that evaluation of snow-avalanche measurement data will yield errors in the range of the presented errors, i.e., between 2 m and 5 m, see Fig. 14b. Compared to typical travel distances of particles in snow avalanches, which are between 115 m and 575 m for a snow avalanche with 23 s duration [51], the relative position error is 0.35% to 4.35%.

**Table 5:** Maximum and average error in position prior and subsequent to optimization for the experiment with simultaneous translation and rotation for sensors $S_{1}$–$S_{5}$

value		unit	$S_{1}$	$S_{2}$	$S_{3}$	$S_{4}$	$S_{5}$
nonopt.	max($\mathrm{p}_{\mathrm{err}}$)	m	73.070	57.491	90.088	99.031	64.994
	average($\mathrm{p}_{\mathrm{err}}$)	m	19.629	17.492	23.588	26.006	18.809
optimized	max($\mathrm{p}_{\mathrm{err}}$)	m	3.989	4.714	2.357	3.395	4.966
	average($\mathrm{p}_{\mathrm{err}}$)	m	2.165	2.548	1.244	1.520	2.711

### Sensor calibration

In this section we show IMU measurement data prior and subsequent to calibration on the example of IMU $S_{2}$.

#### Gyrometer calibration

**Fig. 15:**
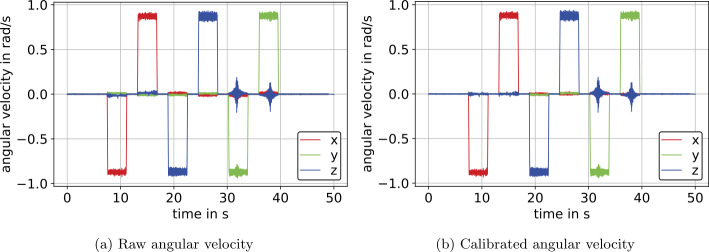
Angular velocity prior and subsequent to the angle-domain calibration on the example of IMU $S_{2}$ (Color figure online)

The application of the angle-domain calibration, see Sect. 4.2, on raw data from the measurement described in Sect. 5.4 yields calibrated angular velocity as shown in Fig. 15b. The associated raw data is displayed in Fig. 15a. For the example of IMU $S_{2}$, the computed calibration matrix and bias are, respectively,

74 $$ {_{\omega }\mathbf{C}} = \left[\textstyle\begin{array}{rrr} {1.00915106} & {-0.02059929} & {0.01363541} \\ {0.01371188} & {1.00421565} & {-0.00048151} \\ {-0.01674715} & {-0.00008789} & {1.00826100} \end{array}\displaystyle \right] , $$

75 $$ {_{\omega }\mathbf{b}} = \begin{bmatrix} -1.27108\times 10^{-2} & -1.58370\times 10^{-2} & 1.09948\times 10^{-2} \end{bmatrix} ^{\mathrm{T}}~\text{rad}\,\text{s}^{-1}, $$

and were derived utilizing the angle-domain calibration from Sect. 4.2.

Note that the $z$-axis components, while rotating about the $y$-axis, see Fig. 15, result from the manipulator crossing a singularity. Comparing the values near zero from Fig. 15a and Fig. 15b, we can see an improvement regarding nonorthogonality. However, more accurate calibration may be derived considering additional rotations in the opposite direction instead of only three rotations, as in the presented calibration method. The scaling error is in the subpercent range, as shown in the diagonal of Eq. (74).

#### Accelerometer calibration

**Fig. 16:**
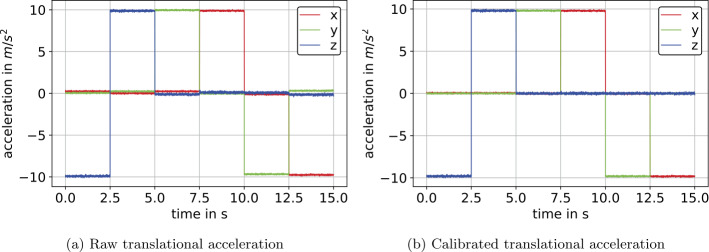
Translational acceleration prior and subsequent to the six-position calibration on the example of IMU $S_{2}$ (Color figure online)

The raw data derived from the six-position calibration measurement, see Sect. 5.4, is shown in Fig. 16a. Applying the calibration, derived in Sect. 4.3, yields angular velocities according to Fig. 16b. The calibration parameters associated to IMU $S_{2}$, and thus Fig. 16, were derived by applying the calibration from Sect. 4.3 yielding a calibration matrix

76 $$ {_{\mathrm{a}} \mathbf{C}} = \left[\textstyle\begin{array}{rrr} 0.99752299 & -0.01784329 & 0.01199042 \\ 0.01783147 & 0.99824794 & -0.00975371 \\ -0.01338447 & 0.00995222 & 0.99040812 \end{array}\displaystyle \right] , $$

and bias

77 $$ {_{\mathrm{a}} \mathbf{b}} = \begin{bmatrix} -8.27747\times 10^{-2} & -1.48133\times 10^{-1} & 1.68094\times 10^{-2} \end{bmatrix} ^{\mathrm{T}}~\text{m}\,\text{s}^{-2}. $$

## Conclusion

Due to stochastic and deterministic errors of IMU measurement data, the deviation of the computed position and orientation increases with measurement duration. Therefore, we proposed a novel optimization method for motion reconstruction of a rigid body. This method corrects measured acceleration and angular velocity by means of correction polynomials, such that the deviations in constraints at the end of motion are minimized. To test the performance of the optimization, two experiments, each with a duration of 23 seconds, were conducted using an industrial manipulator. The two experiments, one translational motion at constant orientation and one motion with simultaneous translation and rotation, were measured by five individual calibrated IMUs of the same type successively. Immediately prior to the experiments, each IMU was calibrated by applying the six-position calibration and the angle-domain calibration to the accelerometer and gyrometer, respectively. As we utilized an industrial manipulator, misalignment between accelerometer and gyrometer is corrected with respect to a common coordinate system. The derived motion-reconstruction method and optimization method were then applied to the measurement data of the five IMUs. A comparison of results prior and subsequent to optimization provided convincing evidence in favor of the presented optimization. For the experiment at constant orientation, the optimization yielded an average maximum position error decrease of 90% for five IMU measurements. Moreover, optimization of simultaneous translation and rotation decreased the average maximum position error by 95%. In addition, the average position error for the experiment at constant orientation and the experiment with rotations decreased by, respectively, 86% and 93%. Thus, the results utilizing the proposed methods contribute significantly toward the minimization of trajectory deviations in inertial-motion reconstruction. Future research will be conducted regarding the application of these methods to snow-avalanche measurement data. Additionally, this method does not prevent sensor fusion; hence a combination may lead to even more accurate motion reconstruction, e.g., applying the derived optimization method to Kalman-filtered data.

## Appendix A: Constraint compliance

In this paper, optimization is utilized to minimize deviations of computed quantities with respect to constraints. Thus, constraints are satisfied to a certain extent. The remaining deviation for the experiment at constant orientation and the experiment with simultaneous translation and rotation are, respectively, shown in Table 6 and Table 7.

**Table 6:** Terminal velocity and position for the experiment at constant orientation for sensors $S_{1}$–$S_{5}$. ${\mathbf {v}_{n}}$ and ${\mathbf {p}_{n}}$ denote velocity and position prior to optimization, respectively. $\mathbf{v}_{\mathrm{opt},\mathrm{n}}$ and $\mathbf{p}_{\mathrm{opt},\mathrm{n}}$ are derived terminal velocity and position, respectively, utilizing the proposed optimization method

value	unit	$S_{1}$	$S_{2}$	$S_{3}$	$S_{4}$	$S_{5}$
$\Vert {\mathbf {v}_{n}}\Vert _{2}$	m s^−1^	0.23	0.53	0.22	0.56	0.56
$\Vert {\mathbf {p}_{n}}\Vert _{2}$	m	3.15	4.16	5.17	8.70	6.82
$\Vert \mathbf{v}_{\mathrm{opt},\mathrm{n}} \Vert _{2}$	m s^−1^	4.80 × 10^−9^	5.59 × 10^−9^	6.12 × 10^−9^	6.17 × 10^−9^	8.47 × 10^−9^
$\Vert \mathbf{p}_{\mathrm{opt},\mathrm{n}}\Vert _{2}$	m	8.20 × 10^−9^	7.67 × 10^−9^	4.52 × 10^−9^	2.84 × 10^−9^	9.30 × 10^−9^

**Table 7:** Terminal velocity and position for the experiment with simultaneous translation and rotation for sensors $S_{1}$–$S_{5}$. ${\mathbf {v}_{n}}$ and ${\mathbf {p}_{n}}$ denote velocity and position prior to optimization, respectively. $\mathbf{v}_{\mathrm{opt},\mathrm{n}}$ and $\mathbf{p}_{\mathrm{opt},\mathrm{n}}$ are derived terminal velocity and position, respectively, utilizing the proposed optimization method

value	unit	$S_{1}$	$S_{2}$	$S_{3}$	$S_{4}$	$S_{5}$
$\Vert {\mathbf {v}_{n}}\Vert _{2}$	m s^−1^	7.58	5.25	10.62	11.54	5.79
$\Vert {\mathbf {p}_{n}}\Vert _{2}$	m	73.07	57.49	90.09	9.90 × 10^1^	64.99
$\Vert \Delta \pmb{\theta}(\mathbf{R}) \Vert _{1}$	rad	8.95 × 10^−2^	1.03 × 10^−1^	1.32 × 10^−1^	1.09 × 10^−1^	3.75 × 10^−2^
$\Vert \mathbf{v}_{\mathrm{opt},\mathrm{n}} \Vert _{2}$	m s^−1^	2.70 × 10^−9^	1.77 × 10^−8^	4.71 × 10^−9^	6.13 × 10^−9^	3.42 × 10^−9^
$\Vert \mathbf{p}_{\mathrm{opt},\mathrm{n}}\Vert _{2}$	m	7.95 × 10^−10^	8.47 × 10^−9^	1.15 × 10^−9^	5.43 × 10^−9^	2.22 × 10^−9^
$\Vert \Delta \pmb{\theta}(\mathbf{R}_{\mathrm{opt}}) \Vert _{1}$	rad	7.55 × 10^−8^	8.11 × 10^−8^	6.82 × 10^−8^	3.22 × 10^−3^	1.00 × 10^−7^

## Appendix B: Correction coefficients

Mean value $\mu $ and standard deviation $\sigma $ for correction coefficients of sensors $S_{1}$–$S_{5}$ are derived by means of Numpy functions numpy.mean and numpy.std, respectively, for the experiment at constant orientation (Table 8) and simultaneous translation and rotation (Table 9).

**Table 8:** Mean value and standard deviation of correction coefficients for the experiment at constant orientation

value	*μ*	*σ*
_0_**c**	8.600 × 10^−3^	1.713 × 10^−2^
_1_**c**	−1.035 × 10^−3^	9.604 × 10^−4^

**Table 9:** Mean value and standard deviation of correction coefficients for the experiment with simultaneous translation and rotation

value	*μ*	*σ*
_0_**c**	5.185 × 10^−3^	1.297 × 10^−1^
_1_**c**	−2.235 × 10^−4^	8.854 × 10^−3^
_*ω*_**c**	−2.839 × 10^−4^	3.284 × 10^−3^

## Footnotes

1 In this work, $^{\textrm {I}}\mathbf{g}$= 9.805531 m s^−2^, which is the gravitation for Innsbruck according to [36].

2 In [16], the authors compared the explicit Euler method with the trapezoidal rule, and neither method had a significant advantage over the other.

3 Constraint definition is indicated by the ($\overset{!}{=}$) sign.

4 Sensor settings: accelerometer range: ${\pm}16~\text{g}$; gyrometer range: ${\pm}2000^{\circ}/\text{s}$.
